# Psychosocial factors for influencing healthy aging in adults in Korea

**DOI:** 10.1186/s12955-015-0225-5

**Published:** 2015-03-07

**Authors:** KyungHun Han, YunJung Lee, JaSung Gu, Hee Oh, JongHee Han, KwuyBun Kim

**Affiliations:** Behavioral Science Research Center, Korea University, Seoul, South Korea, 136-701, 1, 5-ka, Anam-dong, Sungbuk-ku, Seoul South Korea; College of Nursing, Seoul Women’s College of Nursing, 38 Ganhodae-ro, Seodaemun-gu, Seoul South Korea; College of Nursing Science, Kyung Hee University Seoul, Korea, 1 Hoegi-dong, Dongdaemun-gu, 130-701 Seoul South Korea

**Keywords:** Healthy aging, Depression, Perceived heath status, Self-esteem, Achievement, Ego-integrity, Leisure activities, Loneliness

## Abstract

**Background:**

Healthy aging includes physical, psychological, social, and spiritual well-being in later years. The purpose of this study is to identify the psychosocial factors influencing healthy aging and examining their socio-demographic characteristics. Perceived health status, depression, self-esteem, self-achievement, ego-integrity, participation in leisure activities, and loneliness were identified as influential factors in healthy aging.

**Methods:**

171 Korean adults aged between 45 and 77 years-old participated in the study. Self-reporting questionnaires were used, followed by descriptive statistics and multiple regressions as inferential statistical analyses.

**Results:**

There were significant differences between participants’ general characteristics: age, education, religion, housing, hobby, and economic status. The factors related to healthy aging had positive correlation with perceived health status, self-esteem, self-achievements, and leisure activities, and negative correlation with depression and loneliness. The factors influencing healthy aging were depression, leisure activities, perceived health status, ego integrity, and self-achievements. These factors were able to explain 51.9%.

**Conclusions:**

According to the results, depression is the factor with the greatest influence on healthy aging. Perceived health status, ego integrity, self-achievement, self-esteem, participation of leisure activities were also influential on healthy aging as beneficial factors.

## Background

Every person marks each stage of human development with certain achievements, and each stage is affected by the previous stage while additionally affecting the next one. Quality of life in old age is therefore influenced by the individual’s lifestyle as an adult, and preparation for senescence should be made during adulthood. But because such preparation is crucial in determining the quality of life in old age and other health-related attitudes, relevant education may play an important role even from childhood [1]. Therefore, it is important to investigate the recognition process of healthy aging and identify the influential factors in healthy aging at each stage of life.

Healthy aging is a lifelong process of optimizing opportunities for improving and preserving physical, social and mental wellness, independence, and quality of life, as well as enhancing successful life-course transitions [2]. This definition includes the physical, psychological, social, and spiritual well-being of old adults. It also signals an increasingly positive perspective on elderly health and well-being. In recent studies, certain terms such as active aging, successful aging, positive aging, and productive aging are used interchangeably to indicate healthy aging. For example, Kim and Chung uses “successful aging” synonymously with healthy aging [3,4]. However, while successful aging is more of a goal of old age, healthy aging can be considered as a series of processes for achieving successful aging, and is therefore a concept more pertinent to daily living [5]. Healthy aging is what should be taken into consideration for maintaining autonomy and independence towards successful aging.

Nowadays, current trends indicate a populace unsatisfied with just the basic necessities of life in elderly living (in other words, being merely disease- and disability-free), showing instead a desire to maintain their current lifestyles, complete with all of their usual social and leisure activities. Healthy aging extends beyond the mere absence of disease or infirmity, and now includes physical, mental, and social well-being. The mental health of old people, consequently, also encompasses both positive mental health as well as disease prevention. For this reason, much research on healthy aging related to mental health have focused more on identifying the psychological factors for active or successful aging or for psychological well-being, differing from previous studies that tended to concentrate on mental ill-health [6-8]. Consequently, many studies focus on the beneficial or risk factors for positive mental health, such as depression, self-esteem, self-efficacy, loneliness, and isolation, all factors that influence healthy aging.

In the present study, we propose to examine perceived health status, depression, self-esteem, self-achievement, ego-integrity, participation in leisure activities, and loneliness as affecting factors on healthy aging. According to Mossey and Shapiro, well-being and mortality in old people are influenced by their belief in their own health as being good or poor, and old people who perceived their health more positively show higher well-being and less mortality [9]. Individual psychological resources such as self-esteem, self-achievement, and ego-integrity are also important psychological factors for healthy aging [6,10,11]. Several previous studies additionally indicate that self-esteem is significantly correlated to our life outcomes such as human relationships, work, health, and healthy aging. However, the debate continues as to whether self-esteem is a cause and/or consequence of healthy aging due to causal effect. A sense of self-achievement also increases feelings of self-worth and self-efficacy, improving positive mental health status as well as the ability to have the psychological well-being necessary for healthy aging [11,12]. It seems that old people possess a sense of self-achievement through the participation of social or leisure activities; many studies report leisure activities as a good indicator related to healthy aging [13,14]. Other studies report that participation in leisure activity relates to depression especially in old people [15,16]. Depression negatively correlates with mental health, with some studies reporting depression as a significant risk factor affecting healthy aging [17,18]. Loneliness and isolation among old people may increase due to decreased personal relationships, with such social isolation inducing or exacerbating geriatric depression [19]. This study investigates the ego-integrity affect on healthy aging because of how it can be a completion of psychological well-being in old age, according to Erikson’s psychosocial developmental theory [20]. Erikson’s concept of ego-integrity is complex for it may develop with integrating one’s life experience. Erikson also considered it as the result of seven stages of psychosocial development [20]. According to certain cross-sectional studies, generativity effects ego-integrity as an important predictor, and Erikson’s prior stages are significantly correlated to ego-integrity [21,22]. Torges et al. using a longitudinal dataset investigated how attaining successful generativity in midlife corresponds with higher levels of ego-integrity in later life [23]. Psychological well-being acquired through ego-integrity may also influence healthy aging, but there are fewer studies examining ego-integrity as a factor influencing healthy aging.

All the factors mentioned above affect healthy aging in correlation to each other, but many of the previous studies investigate only one or two of these variables. In the present study, we examined the above psychosocial factors by including varying socio-demographic characteristics and identifying the relative powers between the factors influencing healthy aging, with a final goal of facilitating social and clinical intervention for healthy aging. We also expanded the age range from 45 years-old, unlike previous studies which tend to set their range from 60 or 65, due to studies on the aging process indicating that people tend to commence practical preparation for old age approximately in their 40s [24]. Koreans in particular may tend to be engaged in healthy aging processes at this age due to their early retirement system.

## Method

The present study investigated the psychosocial influence factors concerning the healthy using self-reporting questionnaires examining with descriptive language, followed by an analysis of the answers using inferential statistics.

## Subjects

The study sample consisted of 171 adults, aged between 45 and 77. All participants were volunteers recruited from institutions in the local community such as community centers, welfare centers, and church community programs for adults. These adults were located in Seoul and two other cities in Gyeonggi Province, South Korea. Two proctors visited and collected data at these places, and gave more detailed information about the procedures of the study and asked to provide written informed consent if they wished to enroll. We excluded people who have a history of alcoholism, and people who were diagnosed with any psychiatric illnesses including depression and dementia by a clinician and who were under medical treatment. Regarding chronic diseases such as diabetes and hypertension, we included participants who were taking medication as outpatients but excluded adults who were hospitalized because of relevant diseases and needed the help of a caregiver.

Their demographic characteristics are reported in Table 1. They were recruited according to the convenience and snowball sampling methods. All participants provided informed consent prior to the administration of the procedure. Regarding sample size, a power analysis was conducted using the G*power 3.1 program [25]. Then, a minimum sample size was set for the present study at 153 (Cronbach’s alpha = .05, effect size = .15, power (1-β) = .95, 7 independent variables). Although the minimum sample size required was 153, we collected data from 171 participants based on the expectation of missing data or error responses.

**Table 1:** **Sociodemographic characteristics of respondents**

**Total N = 171**						
**Variables**	**Category**	**N(%)**	**M(SD)**	**t or F**	***p***	**Post-hoc**
**Sex**	Male	74(45.4)	3.344(±0.533)	−0.434	.664	
	Female	97(54.6)	3.365(±0.524)			
**Age (years)**	45-60	66(39.6)	3.230(±0.490)	3.113	.015	b
	61-70	77(45.0)	3.411(±0.543)			b
	>71	28(16.4)	3.381(±0.490)			a
**Education level**	Middle school	60(38.2)	3.279(±0.585)	6.828	.000	a
	High school	85(45.0)	3.347(±0.497)			a
	College or above	26(20.8)	3.519(±0.461)			b
**Religion**	Yes	121(64.1)	3.436(±0.535)	4.682	.000	
	No	50(35.9)	3.210(±0.485)			
**Marital status**	Yes	158(78.9)	3.373(±0.528)	1.393	.163	
	No	13(21.1)	3.290(±0.529)			
**Residential status**	One’s own house	155(77.7)	3.400(±0.510)	4.266	.001	
	Rental house	16(22.3)	3.194(±0.542)			
**Avocation**	Yes	113(61.0)	3.519(±0.503)	9.290	.000	
	No	48(39.0)	3.098(±0.465)			
**Socioeconomic status**	High	9(5.2)	3.749(±0.558)	25.072	.000	a
	Middle	107(62.6)	3.448(±0.472)			b
	Low	55(32.2)	3.167(±0.536)			c
**Income activity**	Yes	102(57.0)	3.394(±0.536)	1.845	.065	
	No	69(43.0)	3.306(±0.516)			
**Medication**	Yes	61(40.2)	3.301(±0.517)	−1.128	.260	
	no	110(59.8)	3.384(±0.550)			

## Measures

### Healthy aging

The Scale for Healthy Aging used for measuring healthy aging was developed by Ko, consisting of 20 items on a 5-point likert-scale [26]. This test measures mainly three different health factors: physical, cognitive-mental, and social-supportive health. The range of scores is between 20 and 100, and a higher score indicates a higher level of healthy aging. The original reliability of the measure is .89 (Cronbach’s α) and it was .88 in the present study.

### Perceived health status

The Health Self Rating was used to measure self-perceived health status. The version used in the current study was normalized for Koreans by Sung and Kim [27], and originally developed by Lawston, Moss, Fulcomer and Kleben [28]. HSR consists of three items on a 5-point scale: 1 item for current health states, another item for health status compared to peer group, and another item for comparing current status to the status of 6 months prior. The reliability of the questionnaire was Cronbach’s α = .79 [27], and the reliability in the study was Cronbach’s α = .86.

### Self-esteem

The normalized Korean version by Jeon of Rosenberg’s Self-Esteem scale was used for measuring self-esteem in the study [29]. This instrument consists of 10 items on a 5-point scale consisting of self-confidence, self-control, and positive self-image. The scale is considered a reliable and valid quantitative tool for self-esteem assessment (Cronbach’s α = .85 by Jeon) and the reliability in the present study was Cronbach’s α = .79.

### Depression

For assessing depression, the Geriatric Depression Scale Short Form-Korea Version (GDSSF-K) normalized for Koreans by Kee was used [30]. This scale was adapted for the purpose of the present study, reconstructed on a 5-point likert scale consisting of yes/no alternative choices for performing regression analysis. The tool is specifically for assessing geriatric depression, consisting of 15 items of the particular symptoms of geriatric depression. The reliability of the tool in the research of Kee was Cronbach’s α = .88 [30], and the reliability in the present study was Cronbach’s α = .86.

### Ego integrity

Ego integrity was assessed using a tool invented by Kim [31] based on the theory of psychosocial development by Erikson. This questionnaire consisted of 31 items on a 5-point scale within 6 subscales: satisfaction of the current life, wise living, attitude of life, acceptance of the past, acceptance of aging, and acceptance of death. The original reliability of the questionnaire is Cronbach’s α = .91 [31], and the reliability in the present study was Cronbach’s α = .92.

### Self-achievement

For assessing self-achievement, we used Roh’s tool [32], consisting of 12 items on a 5-point scale. The higher the score was, the higher the self-achievement. Self-achievement measures the level of the subject’s belief, confidence, and positive expectation of his or her own potential, and concretely how the subject realized his or her own value by satisfying life needs and goals. The reliability verified by Roh was Cronbach’s α = .74 [32], and the reliability in this study was Cronbach’s α = .81.

### Participation in leisure activities

Participation in leisure activities was assessed by Seok’s tool [33], consisting of 9 items on a 5-point scale with 3 different levels: at home with family, sports, and appreciation of art. The reliability of this questionnaire was Cronbach’s α = .81 [33], and the reliability in our study was Cronbach’s α = .83.

### Loneliness

The tool used for measuring loneliness was originally developed by Wythers [34]. The revised version for Koreans used in this study that verified its validity and reliability was developed by Maeng [35]. The tool consists of 26 items on a 5-point scale. In the study, loneliness is a measure of the level of the subject’s negative feeling caused by isolation or lack of companionship, and the failure of seeking personal and social relationships. The reliability of the tool in Maeng’s research was Cronbach’s α = .96 [35], and the reliability of the tool in the present study was Cronbach’s α = .95.

### Data collection

The present study was reviewed and approved by the ethics committee at KyungHee University (KHU 2012-S08) before collecting any data, and all subjects provided informed consent for study participation. We conducted a pilot-survey for selecting the tools, randomly recruiting 50 sample subjects. In the main research, we distributed the self-reported form questionnaires with informed consent after providing a proposal for utilizing the data from the research. We collected 180 samples with 171 samples being used in the study, excluding 9 inadequate samples from statistical analysis. 3 participants skipped one or more page without marking and 2 participants dropped out complaining that the questionnaire was too long and 4 participants responded to questions without sincerity such as making with one number on all questions and/or marking a repeated pattern of numbers.

## Data analysis

The collected data were analyzed using SPSS Statistics 20.0 in accordance with the purpose of the study and the characteristics of the variables. The significance level was set at *P <* 0.05 and post-hoc analyses were performed where appropriate, with an effect size set at .15 for regression analysis.

1. To calculate the differences in healthy aging according to the demographic characteristics of the research subjects, t-tests and analyses of variance were conducted. For the post hoc analysis, Scheffe’s method was used.
2. The healthy aging of the subjects and relevant variables, averages, and standard deviations were measured.
3. For the correlations within the variables related to the healthy aging of the research subjects, Pearson’s product moment correlation coefficient was used to examine the relevance.
4. To examine and compare the factors influencing the healthy aging of the research subjects, a regression analysis was conducted on perceived health status, self-esteem, depression, ego integrity, self-achievement, participation in leisure activities, and loneliness.
5. Internal consistency of the tools was estimated by Cronbach’s alpha reliability coefficients.
6. We divided the participants into 3 groups by age in order to compare age difference: 45–60 years-old, 61–70 years-old, and 71 year-old over. According psychosocial development theories, Erikson sorted 40’s and 50’s into middle age and Havinghust et al. also defined the middle aged to be from 30 to 60 [36,37]. We followed a recent gerontology study’s sub-grouping for our study, which distinguishes the young old (60–69), the middle old (70–79), and the very old (80 and over) recognizing the diversity of old age, especially considering how the retirement age is comparatively earlier in Korea than in European countries or the US (regular retirement starts from 55 years old in Korea) [38].

## Results

### Demographic characteristics and healthy aging

The differences in the healthy aging by demographic characteristics of the research subjects were examined by *t*-test and ANOVA, and Scheffe’s test was used for the post hoc test (see Table 1). As a result, there was no significant difference between sex difference [*t* = −0.43, *p* = .664]. In terms of age, healthy aging scores in the group of people 61–70 years-old was higher than in the group of people of 71 years-old or older, and the scores in the group of 45–60 years-old was lowest [F = 3.11 *p* = .015], but the group of 71 years-old or older differed from the two groups of people 45–60 years-old and 61–70 years-old according to the results of the *post hoc* test. People with higher educational backgrounds showed a higher degree of healthy aging [F = 6.82, p < .001). People with religion showed a higher degree of healthy aging than those without religion [*t* = 4.68, *p* < .001]. No difference was found between married and non-married participants [*t* = 1.39, *p* = .163]. Regarding residential types, home-owning subjects showed a higher degree of healthy aging than those who rent property [*t* = 4.27, *p* = .001]. People with a hobby presented a higher score of healthy aging than those who did not have one [*t* = 9.29, *p* < .001]. People with higher income levels showed having healthy aging [*F* = 2.51, *p* < .001]. Although people who were involved in economic activities showed a higher score of healthy aging [mean = 3.39 ± 0.54], the result was not statistically significant [*t* = 1.85, *p* = .065]. Finally, treatment with medications appears to have no influence on the degree of healthy aging [*t* = −1.13, *p* = .260].

### Correlations between healthy aging and psychosocial factors

The mean score of healthy aging was 3.36 (SD = .53). The means of the other relevant variables were: self-achievement [M = 3.66, SD = .55], self-esteem [M = 3.42, SD = .60], perceived health status [M = 3.28, SD = .68], loneliness [M = 2.93 SD = 0.73], ego integrity [M = 2.85, SD = .28], participation in leisure activities [M = 2.81, SD = .74], and depression [M = 2.54, SD = .64]. We examined the correlation between healthy aging and psychosocial factors for observing the correlation of the factors with each other (see Table 2). In the results, perceived health status [*r* = .448, p < .001], self-esteem [*r* = 0.53, p < .001], self-achievement [*r* = 0.44, p < .001], and participation in leisure activities [*r* = 0.54, p < .001] had significantly positive correlation with healthy aging. Depression [*r* = − .60, p < .001], and loneliness [*r* = − .19, p < .001] were negatively correlated to healthy aging at statistically significant levels. Depression and loneliness negatively affected healthy aging in this study. Thus, depression and loneliness correlated positively with each other but they had negative correlation with other all positive factors in the study. However, we could not observe significant correlation between ego integrity [*r* = − .002] and healthy aging.

**Table 2:** **Correlation coefficients between healthy aging and variables**

	**Healthy aging**	**Perceived health status**	**Self-esteem**	**Depression**	**Ego-integrity**	**Self-achievement**	**Participation of leisure activities**	**Loneliness**
Healthy aging	1.000							
Perceived health status	.449^***^	1.000						
Self-esteem	.525^***^	.368^***^	1.000					
Depression	-.595^***^	-.421^***^	-.711^***^	1.000				
Ego-integrity	-.002	-.061	-.144^**^	.318^***^	1.000			
Self-achievement	.439^***^	.229^***^	.390^***^	-.390^***^	.057	1.000		
Participation of leisure activities	.539^***^	.333^***^	.327^***^	-.409^***^	-.026	.385^***^	1.000	
Loneliness	-.193^***^	-.201^***^	-.280^***^	.427^***^	.402^***^	-.147^**^	-.215^***^	1.000

^***^ : p < .001, ^**^ < .01.

### Psychosocial factors influencing healthy aging

The Stepwise regression analysis was used for examining the psychosocial influence factors of the healthy aging. As a result (see Table 3), healthy aging was shown to be significantly influenced by depression, participation in leisure activities, perceived health status, ego integrity, self-achievement, and self-esteem (in that order). However, loneliness did not seem to affect healthy aging. The explanatory power of the final model was relatively high [R^2^ = .526] and the final regression model was statistically significant [*F* = 91.2, *p* < .001]. In addition, a variable with a higher tolerance limit is better because the tolerance limit is a variance in which the independent variable is not explained by other variables. Independent variables are statically valuable if VIF (variance inflation factor) is less than 3. In the present model, all VIFs were less than 3. We confirmed that the error terms also showed a normal distribution examining the P–P plot of error terms.

**Table 3:** **Factors affecting healthy aging in the study samples**

**Variables**	**B**	**β**	**t**	***p***	**Tolerance**	**VIF**	**Adj. R** ^**2**^ **&** **F**
**A constant**	1.652		5.944	.000			Adj. R^2^ = .526 F = 91.2 (*p* < .000)
**Depression**	−0.273	−0.331	−6.613	.000	0.385	2.599	
**Participation of leisure activities**	0.196	0.273	7.594	.000	0.744	1.344	
**Perceived health status**	0.123	0.158	4.508	.000	0.781	1.280	
**Ego-integrity**	0.242	0.129	3.820	.000	0.846	1.182	
**Self-achievement**	0.113	0.117	3.236	.001	0.741	1.349	
**Self-esteem**	0.101	0.114	2.523	.012	0.469	2.132	

## Discussion

The purpose of the present study was to find the psychosocial factors influencing healthy aging for assisting the preparation of active and successful aging, a socially relevant issue as interest in and demand for healthy aging continues to increase due to the elongation of our average lifespan and the growing elderly population. For this reason, we selected seven psychosocial influence factors of healthy aging suggested by previous studies, observing how these factors affect healthy aging in tandem. A further goal of this study is to promote the development of psychosocial intervention that maintains and promotes healthy life in old age.

As we analyzed demographic characteristics, we found that age, religion, educational background, residential status, economic level, and hobbies were influential in healthy aging, with sex difference, spousal status, participation of income activities, and taking medication as having no effect. Linda suggests that the most influential social factors on healthy aging are family status and religion [5], while some studies report that age, economic level, existence of a spouse, healthy and positive thinking, and hobbies are more important social influencing factors in healthy aging [39,40]. Some of our results were consistent with these previous studies but some results were not, and the results of previous researchers are also not completely coherent. These discrepancies are probably due to differences in the socio-demographic factors of the samples in each study and/or by territorial cultures and the current atmosphere of the samples’ societies.

Most psychosocial influence factors selected in the present study affected healthy aging, according to the results. In the regression analysis, the factors that significantly influenced healthy aging were determined to be depression, participation in leisure activities, perceived health status, ego integrity, self-achievement, and self-esteem (in that order). These results were consistent with previous studies reporting that depression negatively influences healthy aging [41,42]. In effect, several studies reveal a similar result with our findings. Some studies report that a higher perceived health status results in higher healthy aging [43-45], and another study points out the importance of psychological well-being on healthy aging [46], while still other studies suggest that self-achievement and self-esteem relate to healthy aging [47,48].

In our study, depression was a more influential psychological factor on healthy aging. This finding means that not only physical health but also mental health is very important to healthy aging and the life of old people. Notably, Johnson and Barer report that people with good mental health are conscious of successfully aging, even though their physical health status is considered relatively poor [49]. In effect, many studies reveal that good mental health can reduce depression as well as a sense of alienation and loneliness by enhancing self-esteem due to increasing their participation in social activities [40,43]. Old people tend to enjoy their daily lives more by maintaining their mental health and vitality of life by doing leisure activities for stimulation and self-development even if their activities are focused exclusively on work or income, and this is a trend of healthy aging. However, loneliness did not influence healthy aging, and we could not find research that suggested any direct influence, even though several researchers report that loneliness is related to mental health and cognition, and is also predictive of depressive symptoms. In our research, loneliness might be influenced by depression during the regression analysis or simply was not a primary influence factor on healthy aging.

Similar to previous studies, perceived health status, self-esteem, self-achievement, ego-integrity, and participation in leisure activities positively affected healthy aging [6,9,11,43-45,47,48]. Hong pointed out that psychological well-being is very important in healthy aging and for this reason, the psychological perspective, the psychosocial, and the multidimensional model of healthy aging are regarded with more attention in recent healthy aging studies [46,50-54]. Belief in one’s own health as being good or poor is very important for healthy aging. Mossey and Shapiro report that old people with more positive perceived health show higher well-being and less mortality, while other studies report a positively perceived health status affects healthy aging, consistent with our results [9,43-45]. The variables regarding individual psychological resources such as self-esteem, self-achievement, and ego-integrity also consistently influence healthy aging in our findings as in previous studies [6,10,11]. However, we cannot know for sure whether self-esteem is a cause and/or consequence of healthy aging due to causal effects, even though many studies report that self-esteem influences healthy aging. Self-esteem correlates significantly to our life outcomes in all age groups, not only for old age. Not surprisingly, self-achievement also affects healthy aging, as in previous studies [11,12]. The experience of achievement may improve positive mental health status, but old people have less opportunity to experience achievement. Participation in social or leisure activities may enable a sense of self-achievement; leisure activities was a positive factor on healthy aging, an observation consistent with previous studies, with some studies reporting that participation in leisure activities decreases depression particularly in old people [13-16]. As our results show, all individual psychological resources correlate to social integration because old people strengthen and improve their individual psychological resources through participating in social and leisure activities. However, as mentioned before, old people tend to have less of a chance to participate, especially after retirement. More public concern for increasing the opportunities for their participation in social and leisure activities is needed, in addition medical services and financial support.

Notably in our investigation, ego-integrity was included as an influencing factor by regression analysis, but it did not significantly correlate with healthy aging. According to many previous studies, ego-integrity is related to the factors of health status, social activities, self-esteem, self-efficacy, and social support, as well as other later life adjustment indexes such as life satisfaction, death anxiety, and sleep disturbance [55-58]. Consistently in our results, ego-integrity correlated significantly with psychological factors such as depression, loneliness, and self-esteem but not to perceived healthy status, participant leisure activities, and self-achievement. According to our findings, ego-integrity may have an effect on healthy aging, but not directly (or primarily) because healthy aging is inevitably influenced by physical health in spite of the importance of mental health. However, it is obvious that attainment of ego-integrity is an essential task for old people as Guzman et al. mentioned because ego-integrity is a more stable factor that reflects the status of psychological well-being in later life, which is influenced by one’s entire prior life according to Erikson’s psychosocial development theory [59]. Erikson and colleagues already reported that successful balancing of a development stage assists in facilitating the subsequent stage [60]. And many studies using both cross-sectional and longitudinal data report that achieving successful generativity in mid-life facilitates attaining high levels of ego-integrity in later life [21-23]. Ego-integrity does seem to influence stability in our study as well. But there are certain limits to discussing with our results how ego-integrity affects healthy aging, and therefore it would be worthwhile to observe how ego-integrity affects healthy aging independently through studies with diverse aspects.

There was no single-factor determinant of healthy aging. Many previous studies independently investigated healthy aging through biomedical or psychological perspective studies [50,51]. Nowadays, the multidimensional model of healthy aging is generally accepted because the factors of both perspectives are related reciprocally even though they are theoretically dissimilar [52-54]. Interestingly, we can observe similar outcomes in economically developed countries in both the East and the West. Ng et al. report that a multidimensional definition of successful aging identifies more than the biomedical definition in a Chinese-Singaporean sample that analyzes both cross-sectional and longitudinal data, and another cross-sectional study with Chinese elderly in Shanghai reported similar results as well [61,62]. According to a systematic review of Cosco et al., they report that physiological factors such as physical status, disability, and disease presence are more effective on healthy aging, but psychosocial factors such as affective status, social relations, and psychological well-being are more salient similarly in all countries from studies analyzing 84 research articles published in between 2011 and 2013 in developed European, American, and Asian countries including South Korea [63]. Koreans generally had a negative perspective of retirement and aging because in the past, Korean old people tended to be physically and economically dependent on their children and had to live together. Therefore, their scope of life and social activities were relatively limited. In the last decade, the perception of retirement and aging has changed more for the positive, but not as much as to the level of Westerners. According to a research report in 2010 from The Korea Institute for Health and Social Affairs, Korean old people spend more money on leisure activities, medical care, and exercise not only for maintaining physical status and avoiding diseases, but also for positive life-experiencing achievement, improving social relationships, avoiding depressive feelings, and integrating into society [64]. Recent studies from South Korea on healthy aging emphasize the importance of psychosocial factors including ego-integrity, similarly to our study’s outcome, which were not regarded as important in studies on healthy aging in the 90s [3,4,6,7,11].

The maintenance of high levels of physical function is still an important indicator of healthy aging. The level of physical activity and the functional status in old people correlates with psychosocial variables such as depression, psychosocial skills, self-efficacy, and social support [65]. Some psychosocial factors for influencing healthy aging that are found in our study, such as depression, perceived health status, and participation in leisure activities, are related to physical activities in old adults. Several studies show that depression and poor perceived health status are risk factors in functional status decline in old people, and old people who have only a single chronic disease or were dependent on others for caregiving had low self-esteem and experienced more depressive feelings in their daily lives [66,67]. Inevitably, the status of physical function in old people was a more basic fundamental factor on healthy aging interacting with psychological factors and built environmental factors on healthy aging. The strength of social relationships of old people is related reciprocally to the status of physical function and psychological factors such as depression and self-concept [68]. Old people may experience successful achievement, expand their social and personal relationships, have more chance to increase their physical activities participating in leisure activities, and consequently have less depression leading to more vitality in life. According to the outcomes of previous studies, it is true that psychosocial, physical, and environmental factors interact reciprocally with healthy aging [65,69,70].

A major limitation of the study has to do with the research sample and sample-size. It is still difficult to say that our subject group is representative of Korean old people as almost all of the participants live in major cities, giving them more access to various socio-cultural benefits or support than people who live in smaller cities or rural areas. Although the sample-size was satisfactory under statistical analyses for examining the factors affecting healthy aging, a still bigger sample is needed for observing the difference or alteration of psychosocial factors for healthy aging according to age, sex, or other demographic factors. Although this study examines the significant correlation between influential psychosocial factors and healthy aging, there are limitations to making strong causal conclusions and in investigating mediating effects between the factors due to the cross-sectional design of study. Another limitation is that the variables assessed in our study are not very novel, with the exception of ego-integrity. In future research, other psychological factors that have not been assessed yet in recent healthy aging studies should be examined, as well as the risk factors on healthy aging including more negative psychological factors. Additional research that directly compares biomedical and psychosocial factors on healthy aging are also needed, as the subject’s functional capacity and health behaviors were not measured in the present study.

## Conclusion

The present study investigates the psychosocial factors influencing healthy aging for promoting healthy living in old age and developing socio-cultural care and support for old people. In light of the results, it can be inferred that depression is the factor with the greatest influence on healthy aging. Factors such as participation in leisure activities, perceived health status, ego-integrity, self-achievement, and self-esteem also affect healthy aging with significant correlations between them. In particular, ego-integrity’s influence in completing psychological well-being in old age (according to Erikson’s psychosocial developmental theory) is an interesting finding of this study, meriting further investigation in the future. Finally, our findings make a step toward understanding healthy aging in terms of the psychosocial perspective, and may help both society and individuals in the planning of healthcare for old people.
